# Decreased production of class-switched antibodies in neonatal B cells is associated with increased expression of miR-181b

**DOI:** 10.1371/journal.pone.0192230

**Published:** 2018-02-01

**Authors:** Stephanie Glaesener, Christine Jaenke, Anika Habener, Robert Geffers, Petra Hagendorff, Katrin Witzlau, Esther Imelmann, Andreas Krueger, Almut Meyer-Bahlburg

**Affiliations:** 1 Department of Pediatric Pneumology, Allergy and Neonatology, Hannover Medical School, Hannover, Germany; 2 Biomedical Research in Endstage and Obstructive Lung Disease (BREATH), Member of the German Center for Lung Research (DZL), Hannover, Germany; 3 Genome Analytics, Helmholtz Centre for Infection Research, Braunschweig, Germany; 4 Institute of Immunology, Hannover Medical School, Hannover, Germany; 5 Institute for Molecular Medicine, Goethe University, Frankfurt am Main, Germany; Monash University, AUSTRALIA

## Abstract

The increased susceptibility to infections of neonates is caused by an immaturity of the immune system as a result of both qualitative and quantitative differences between neonatal and adult immune cells. With respect to B cells, neonatal antibody responses are known to be decreased. Accountable for this is an altered composition of the neonatal B cell compartment towards more immature B cells. However, it remains unclear whether the functionality of individual neonatal B cell subsets is altered as well. In the current study we therefore compared phenotypical and functional characteristics of corresponding neonatal and adult B cell subpopulations. No phenotypic differences could be identified with the exception of higher IgM expression in neonatal B cells. Functional analysis revealed differences in proliferation, survival, and B cell receptor signaling. Most importantly, neonatal B cells showed severely impaired class-switch recombination (CSR) to IgG and IgA. This was associated with increased expression of miR-181b in neonatal B cells. Deficiency of miR-181b resulted in increased CSR. With this, our results highlight intrinsic differences that contribute to weaker B cell antibody responses in newborns.

## Introduction

The immaturity of the developing immune system in early life is reflected by an increased susceptibility to infections and decreased vaccination responses. Multiple factors within the innate and adaptive arm of the immune system have been identified contributing to this immaturity. Regarding humoral immunity, the neonate is initially protected by passively acquired maternal antibodies, although neonates are able to mount T cell-dependent and—independent immune responses [1–3]. However, neonatal antibody responses are delayed in onset, show decreased peak levels, are of shorter duration, and of lower antibody affinity [3–5]. The generation of high-affinity, class-switched, antibodies is a complex process relying on the interaction of T-, B-, and antigen-presenting cells (APCs). Thus, the immaturity of the neonatal immune system results from quantitative and qualitative deficiencies of many different cell types and their interplay with each other.

Neonatal APCs are found to be immature in terms of both number and function: The number of CD11c^+^ DC in mice and humans was reduced in neonates [6, 7], and neonatal DC were deficient in upregulating costimulatory molecules including MHC-II, CD80, and CD86 upon stimulation, indicating their inability to fully activate antigen specific T and B cell responses [8–10].

Within the T cell compartment, lower numbers of effector-memory and follicular helper T cells were described, thereby impacting the generation of high-affinity antibodies [11]. In addition, production of IL-4 and IL-21, as well as the expression of surface CD154 were shown to be reduced in activated cord blood T cells [12–15].

Regarding B cells, previous studies have shown that the peripheral B cell compartment notably differs between newborns and adults. Most importantly, CD27^+^ memory B cells are almost completely missing in neonates due to the lack of previous antigenic exposure [16–18]. Moreover, the CD27^-^ naïve B cell fraction in neonates consists predominantly of transitional 1 and 2 (T1 and T2) B cells that are recent bone marrow emigrants [19], and only few mature naïve B cells. In addition, several phenotypic differences between neonatal and adult B cells have been described: Neonatal B cells have been shown to express different levels of various cell surface molecules, including CD22, CD40, CD80, CD86, and IgM [14, 20–23]. Functional differences were identified regarding cytokine secretion, antigen presentation, and a decreased ability of neonatal B cells to class-switch [7, 8, 15, 24, 25].

Epigenetic mechanisms play important roles in developmental processes of many cell types. In particular, B cell development, activation and differentiation are regulated by miRNAs through repressing gene expression and impacting post-transcriptional processes [26–29]. Based on these data, impaired antibody responses in neonates seem to be the combined result of an altered microenvironment, a different composition of B cell subsets towards a more immature phenotype and B cell intrinsic factors. The latter are, especially in humans, still barely defined, and their influence remains vague. We therefore phenotypically, and functionally, characterized distinct B cell subpopulations from neonatal cord and adult peripheral blood. When directly comparing corresponding B cell subsets, no phenotypical variances except for IgM expression were found between neonates and adults. In contrast, functional analyses revealed significant differences in proliferation, B cell receptor (BCR) signaling and immunoglobulin (Ig) production. Furthermore, differential expression of microRNA-181b (miR-181b) was identified as one important factor for impaired class-switching of neonatal B cells. In summary our data demonstrate that neonatal B cells are functionally intrinsically immature.

## Materials and methods

### Antibodies and reagents

Detailed information about used antibodies, staining panels, and primers for human and murine B cell immunophenotypic characterization are shown in S1, S2 and S3 Figs. All Kits were used according to the manufacturer´s recommendations.

### Cell preparation, flow cytometry and cell sorting

PBMC were isolated from heparinized cord blood of neonates (obtained from the Clinic for Gynecology and Obstetrics, Medical School Hannover, Germany), from leukocyte filters of anonymous healthy adult donors (age range 18–65 years) obtained from the Institute for Transfusion Medicine, Medical School Hannover, Germany), or from freshly drawn blood from healthy individuals (age range 20–55 years) by density gradient centrifugation using Biocoll (Biochrom, Berlin, Germany) as described earlier [30]. Samples were obtained after informed consent of the individuals (written consent from cord blood donors) in accordance with the Declaration of Helsinki, and ethical approval (No. 5240) was obtained from the local ethics committee (Hannover Medical School, Hannover, Germany). EasySep Human B cell Enrichment Kit w/o CD43 Depletion (StemCell Technologies, Grenoble, France) was used to isolate B cells from PBMC by negative selection according to the manufacturer´s protocol.

For multicolor flow cytometry, PBMC were stained with relevant antibodies, acquired on a FACSCanto II (BD Biosciences), and analyzed using FlowJo software (TreeStar). For sorting of B cell subpopulations, B cells were gated as shown in S4 Fig on a FACSAria (BD Biosciences). Cross-contamination of sorted B cells subsets was excluded by staining for IgM and IgD expression (S5 Fig).

### Cell culture and *in vitro* stimulation

Purified B cells were cultured in complete medium (25.000 cells/well; RPMI1640, 10% FCS, 1% HEPES, 1% penicillin-streptomycin, 1% L-glutamine (all Biochrom), and 100μM ß-mercapthoethanol (Invitrogen)). Stimulation agents were added alone or in various combinations at the following final concentrations: CpG-ODN2006 (CpG-oligodeoxynucleotides; CpG; 5μM), anti-IgM/G/A (10 μg/ml), IL-4 (10 ng/ml), IL-21 (50 ng/ml), BAFF (0.5 μg/ml), and anti-CD40 (1 μg/ml).

### Measurement of Ca^2+^-Flux and phosflow

Measurement of *in vitro* cell activation was performed as previously described [31]. Ca^2+^-Flux was measured after stimulation with anti-IgM by flow cytometry using Indo-1 (ebioscience) and 1x10^6^ surface-stained B cells. The relative concentration of intracellular free Ca^2+^ was determined at the median fluorescence ratio of Indo-1 bound/unbound (405/488 nm) over time. For phosflow analysis 1x10^6^ surface-stained B cells were stimulated with anti-IgM/G/A for different periods of time followed by immediate fixation, permeabilization, and subsequent intracellular staining for pTyr.

### Survival and proliferation assay

Sort-purified B cells were stained with carboxyfluorescein succinimidyl ester (CFSE; Life Technologies). Survival and proliferation were analyzed by flow cytometry at indicated time points by gating on live cells (forward-sideward scatter) for survival and measuring CFSE dilution for proliferation.

### ELISA

Quantification of IgM, IgG and IgA was performed by ELISA established in our lab. Briefly, capture antibody-coated microtiter plates were blocked (PBS/0.05%Tween-20/2%BSA) and incubated first with cell culture supernatants or Ig standards, followed by secondary mAb. For development 3,3’,5,5’-Tetramethylbenzidine (TMB) substrate was used. Absorbance was read at 450nm (GloMax^®^-Multi Detection System, Promega).

### Cell cycle-analysis

Cell cycle analysis was performed using a slightly modified protocol previously described by Meyer-Bahlburg et al. [32]. Briefly, 1x10^6^ purified, surface-stained B cells were fixed and permeabilized as described above; DAPI (4’,6-diamidino-2-phenylindole; 1 μg/ml; Roth) and PyroninY (Sigma-Aldrich) were added and samples were acquired by flow cytometry.

### Quantification of KREC

Kappa-deleting recombination excision circles (KREC) were determined as described by van Zelm et al [33] using the 7500 Real-Time PCR System from Applied Biosystems (Foster City, CA, USA). For DNA isolation from B cells we used the Invisorb Spin Micro DNA Kit (STRATEC).

### Analysis of microRNA (miRNA; miR)

Total RNA was isolated from sort-purified T1, T2 and naïve mature B cells obtained using the *mi*rVana^™^ miRNA Isolation Kit (ambion, ThermoFisher Scientific), according to the manufacturer´s protocol. For microarray analysis, RNA from 7 independent neonatal and adult donors was pooled. The screening for differentially expressed miRNAs was performed by using GeneChip^®^ miRNA 2.0 Arrays (Affimetrix, CA, USA) and the Qlucore Omics Explorer 3.0 (Qlucore AB, Lund, Sweden).

For miR181b qPCR we used the following reagents: cDNA: Taqman^®^ small RNA reverse transcription assay; qPCR: TaqMan Universal Master Mix (both Life Technologies). cDNA was synthesized from 10 ng RNA of the above mentioned 7 single donors and qPCR was performed with primers for miR-181b, and as housekeeping control snRNA U6 (S3 Fig).

#### Western blot

B cells were lysed at indicated time points in RIPA buffer (ThermoFisher). Lysates were run on a 10% SDS gel and transferred by semi-dry blotting. Blots were probed with anti-AID followed by anti-β-Actin.

### Mouse experiments

C57BL/6J mice (CD45.2; initially purchased from Charles River) with a targeted deletion in miR-181a/b-1 (miR-181a/b1^-/-^ mice; ko mice) and heterozygous miR-181a/b-1^+/-^ mice (het mice) were generated in the lab of Prof. Krueger, bred, and maintained, in the SPF animal facility of Hannover Medical School and handled according to IACUC approved protocols (Permit: 33.9-42502-04-12/0869, 07/1393, 08/1480) [34]. Neonatal mice were not older than 5 days whereas adult mice were older than 8 weeks (8–16 weeks).

Mice were sacrificed by CO_2_ inhalation and cervical dislocation. Murine splenocytes were mashed through 70μm mesh, centrifuged at 320g for 5 min, counted and resuspended in complete medium (RPMI1640, 10% FCS, 1% HEPES, 1% penicillin-streptomycin, 1% L-glutamine (all Biochrom), and 100μM ß-mercapthoethanol (Invitrogen)). Stimulation of splenocytes was performed for 5d with LPS alone (10 μg/ml) or in combination with CpG (1μM), anti-CD40 (3 μg/ml), and IL-4 (10 ng/ml).

Mouse-specific ELISA assays, for the analysis of serum and cell culture supernatants, and qPCR of miR181b were established and run in the same way as described above for human samples, except for using the Nucleospin^®^Spin RNA Isolation Kit (Macherey-Nagel) and the Maxima SYBR Green qPCR Master Mix (ThermoFisher). The following primers were used: “Mir181b-1 (mouse) qSTAR miRNA primer pair Kit” from Origen for detection of miR181b (Rockville, MD, USA), *Aicda*, and as housekeeping gene *GAPDH* (detailed primer sequences are shown in S3 Fig). For flow cytometry splenic B cells were surface stained and gated as seen in S9 Fig.

### Statistical analysis

All statistical analysis was performed by using GraphPad Prism5, Version 5.02 (San Diego, CA, USA). 1way ANOVA with post-hoc Bonferroni test and Students *t*-test were used to verify statistical difference of results between individual subsets. All results are shown as MEAN ± SEM.

## Results

### Phenotypes of neonatal and adult B cells are similar except for IgM expression

The peripheral B cell compartment of neonates consists mainly of immature B cells. Consistent with previous data [17] we primarily detected CD27^-^CD24^++^CD38^++^ transitional (T1 and T2), few CD27^-^CD24^-^CD38^-^ naïve mature B cells, and almost a complete lack of CD27^+^ memory B cells in neonatal cord blood (CB) compared to adult blood (AB) (S4 Fig and Table 1). In addition, differences in expression of surface markers have been described [14, 20]. However, many of the studies investigated total B cells instead of phenotypically distinct B cell subpopulations.

**Table 1:** Relative distribution of B cell subpopulations (n = 8).

	CD19^+^	CD19^+^CD2^+^	CD19^+^CD27^-^			
				CD24^++^CD38^++^	CD24^+^CD38^+^	CD24^-^CD38^-^
[%]	Total	Memory B cells	Transitional & Naïve mature	Transitional 1 (T1)	Transitional 2 (T2)	Naïve mature (N)
**adult blood (AB)**	7.0 (+/-3.0)	20.3 (+/-6.2)	75.6 (+/-6.7)	1.3 (+/-0.5)	3.9 (+/-1.7)	65.2 (+/-22.4)
**cord blood (CB)**	7.1 (+/-2.1)	2.2 (+/-0.9)	95.4 (+/-1.3)	14.2 (+/-3.0)	54.0 (+/-3.7)	29.0 (+/-3.1)

**Fig 1:**
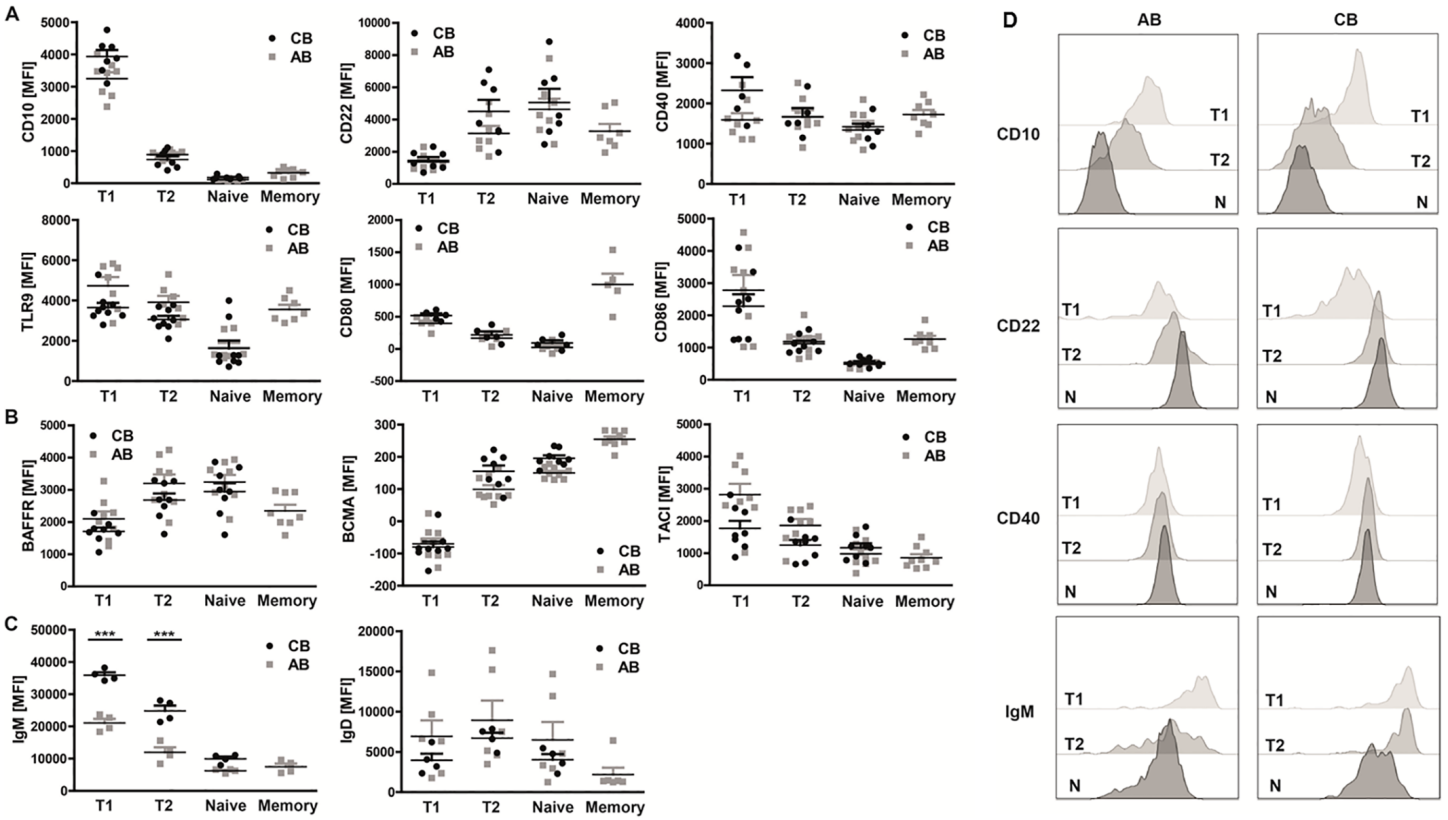
Neonatal and adult B cell subsets share similar phenotypic characteristics. Flow cytometric analysis of surface marker expression of **(A)** maturity (CD10 & CD22; n = 7), co-stimulation (CD40 & TLR9; n≥6/8) and activation (CD80 & CD86; n = 5/3), **(B)** the three BAFF receptors BAFFR (n = 8), BCMA (n = 8/9) and TACI (n = 8/9) and **(C)** of surface IgM (n = 4) and IgD (n = 4/6) on neonatal and adult B cell subpopulations (transitional 1 & 2 B cells: T1 & T2; naïve mature B cells: N), **(D)** Expression of discrete markers on the three populations in AB and CB. Representative of >4 independent experiments are shown. **(A-C)** 1way ANOVA; *** P<0.0001).

Therefore, we carefully determined the expression profiles of a variety of surface molecules including activation markers, costimulatory molecules and the three BAFF receptors BAFFR, BCMA (B cell maturation antigen), and TACI (transmembrane activator and calcium modulator and cyclophilin ligand interactor). All surface molecules were differentially expressed on distinct B cell subsets, but no significant differences could be observed between corresponding neonatal and adult B cell subpopulations (Fig 1A and 1B).

In contrast, IgM, but not IgD, was expressed at a significantly higher level on neonatal compared to adult T1 and T2 B cells (mean fluorescence intensities (MFI) for T1: AB 21070±2542, CB 35907±1767; T2: AB 11985±3030, CB 24830±3338). No difference in IgM expression was observed on naïve B cells (Fig 1C).

### Neonatal T1 and T2 B cells show enhanced proximal BCR signaling

**Fig 2:**
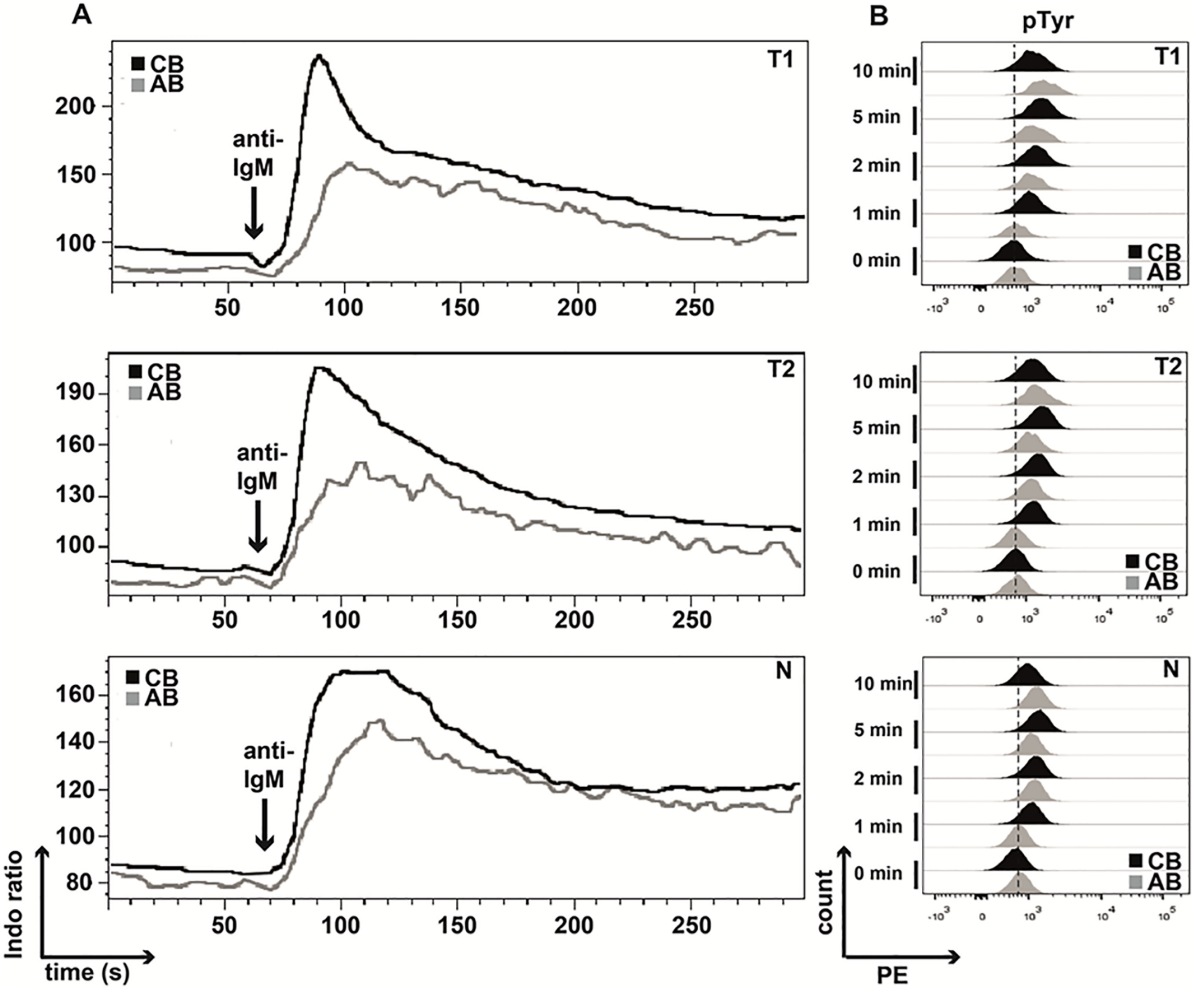
BCR stimulation results in enhanced responses of neonatal B cell subsets. Isolated adult and neonatal B cells were surface-stained for B cell subset discrimination (transitional 1 & 2 B cells: T1 & T2; naïve mature B cells: N) and stimulated via the BCR for flow cytometric determination of **(A)** Ca^2+^-Flux by calculating the Indo-1 ratio measured for 5 min (results of one representative experiment out of 5 are shown), and **(B)** the pTyr status at 1, 2, 5, and 10 min (n = 5). Data of one representative experiment is presented.

While the phenotypical characteristics were comparable between neonatal and adult B cells we subsequently analyzed early BCR signaling events. Determination of calcium levels revealed that all neonatal B cell subsets exhibited higher release of intracellular calcium after stimulation with anti-IgM compared to their adult counterparts (Fig 2A; S6 Fig). Consistently, phosphorylation of tyrosine residues was delayed after BCR triggering in adult B cell subpopulations when compared to their neonatal counterparts (Fig 2B; S7 Fig). Thus, neonatal B cell subsets exhibit enhanced proximal BCR signaling.

### Differences in survival and proliferation between neonatal and adult B cell subpopulations

**Fig 3:**
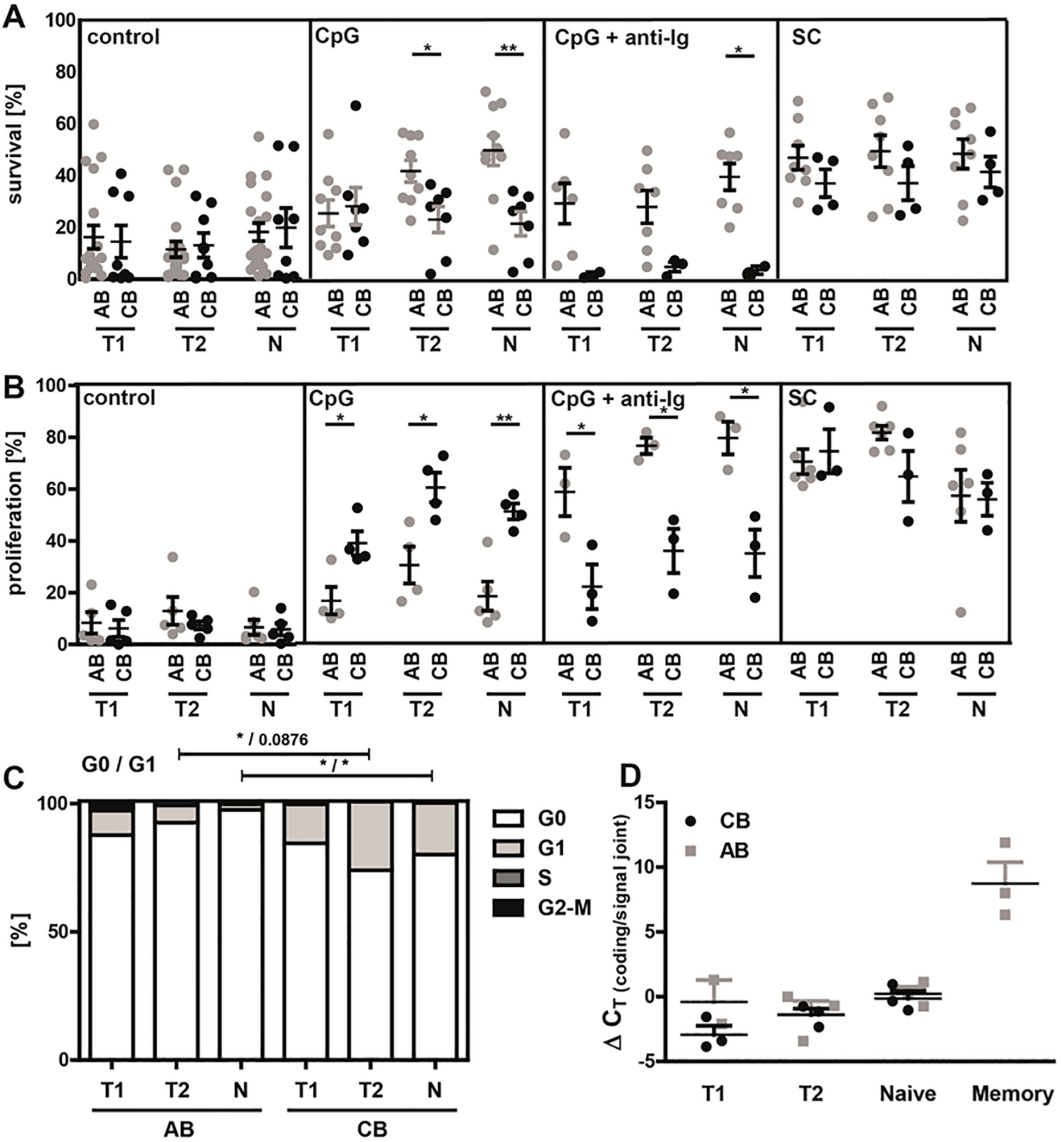
Neonatal B cell subpopulations show different responses in survival and proliferation upon stimulation. Analysis of **(A)** survival (live gate in FSC/SSC dot blot; %) and **(B)** proliferation of CFSE-labeled transitional 1 & 2 (T1 & T2) and naive mature (N) B cell subpopulations of adult blood (AB) and neonatal cord blood (CB) in response to stimulation with either CpG (n≥5), CpG+anti-Ig (n≥3) or to a stimulation cocktail (SC; IL-4, IL-21, anti-CD40, CpG and BAFF; n≥3) for 5 days. As control the cells were cultured with medium alone. **(C)** Distribution of cell cycle phases was analyzed by flow cytometry in adult and neonatal T1, T2 and mature naive B cells by using DAPI and PyroninY (n = 8). **(D)** The replication history of T1, T2, mature naive and memory B cells was determined by using the KREC assay and analyzing the ΔC_T_ of the coding joint and the signal joint PCR from three sorted donor samples. **(A-D)** Students *t*-test; * P<0.05, ** P<0.01.

Our next approach was to test if altered BCR signaling resulted in functional consequences. We therefore determined survival and proliferation in response to a variety of different stimuli: via TLR9 using CpG-ODN alone; CpG in combination with anti-Ig to activate additionally the BCR; and a stimulation cocktail (SC) containing CpG, IL-4, IL-21, anti-CD40, and the B cell survival factor BAFF. Stimulating B cells via TLRs mimics a thymus-independent immune response, resulting in proliferation, few somatic hypermutations and differentiation into IgM memory B cells and plasma cells secreting IgM [35, 36]. Addition of anti-CD40 and different cytokines, imitating cognate T cell help, resembles a more thymus-dependent immune response [37, 38]. To ensure equal starting conditions with regard to cell fitness and to exclude early effects on B cell survival, we determined cell survival by forward-sideward scatter gating at early time points (18h, 30h, and 54h, S8 Fig). No significant differences between neonatal and adult B cell subpopulations were found. Subsequently, survival and proliferation were determined after 5 days of stimulation. Overall, adult B cell subsets showed improved survival compared to their neonatal counterparts when stimulated via TLR9 alone or in combination with anti-Ig (Fig 3A). However, when incubated with the SC neonatal and adult B cells survived equally well.

Analysis of proliferation, as determined by CFSE (carboxyfluorescein succinimidyl ester) dilution in dividing B cells, revealed contrasting results: Stimulation with CpG alone resulted in significantly enhanced proliferation of neonatal B cell subpopulations (T1: AB 20.8±9.1%; CB 44.3±16.0%; T2: AB 37.7±19.8%; CB 57.5±10.9%; Naïve: AB 18.3±14.6%; CB 43.7±13.0%), whereas stimulation with a combination of CpG and anti-Ig led to the opposite result with decreased proliferation of neonatal B cell subsets (T1: AB 58.1±16.2%; CB 21.4±14.9%; T2: AB 75.8±5.4%; CB 35.3±14.8%; Naïve: AB 78.8±10.9%; CB 34.3±15.9%) (Fig 3B). Highest proliferation rates were achieved with the SC without any differences between neonatal and adult B cells. Differences in survival and proliferation in response to CpG were not due to differences in receptor expression as TLR9 was similarly expressed on neonatal and adult B cells (Fig 1A).

Next, we performed cell cycle analysis of freshly isolated B cells using Pyronin Y and DAPI (4’,6-diamidino-2-phenylindole) to measure RNA and DNA content, respectively. No differences were observed comparing neonatal and adult T1 B cells. However, in accordance with previous findings in murine neonatal lymphocytes [39], a significant higher proportion of neonatal T2 and naïve mature B cells was found in G1 phase compared to their adult counterpart (Fig 3C; T2: AB 5.1±2.5%, CB 30.6±25.8%; p = 0.033; Naïve: AB 2.2±2.9%, CB 20.1±21.2%; p = 0.0438).

For the analysis of the proliferation history of B cells we examined KREC (kappa-deleting element recombination circle) levels [33]. The data demonstrate equal numbers of KREC in neonatal and adult B cell subsets (Fig 3D).

Collectively, our data demonstrate significant differences in survival and proliferation between neonatal and adult B cell subpopulations and suggest a slightly pre-activated status of B cells from CB based on cell-cycle analysis. However, neonatal B cells do not have a general proliferative defect.

### Decreased production of class-switched antibodies in neonatal B cells

The secretion of protecting antibodies is the most important function of B cells. We therefore tested *in vitro* the capability of sort-purified neonatal and adult B cell subpopulations to produce Ig’s after stimulation for 8 days using different agents.

**Fig 4:**
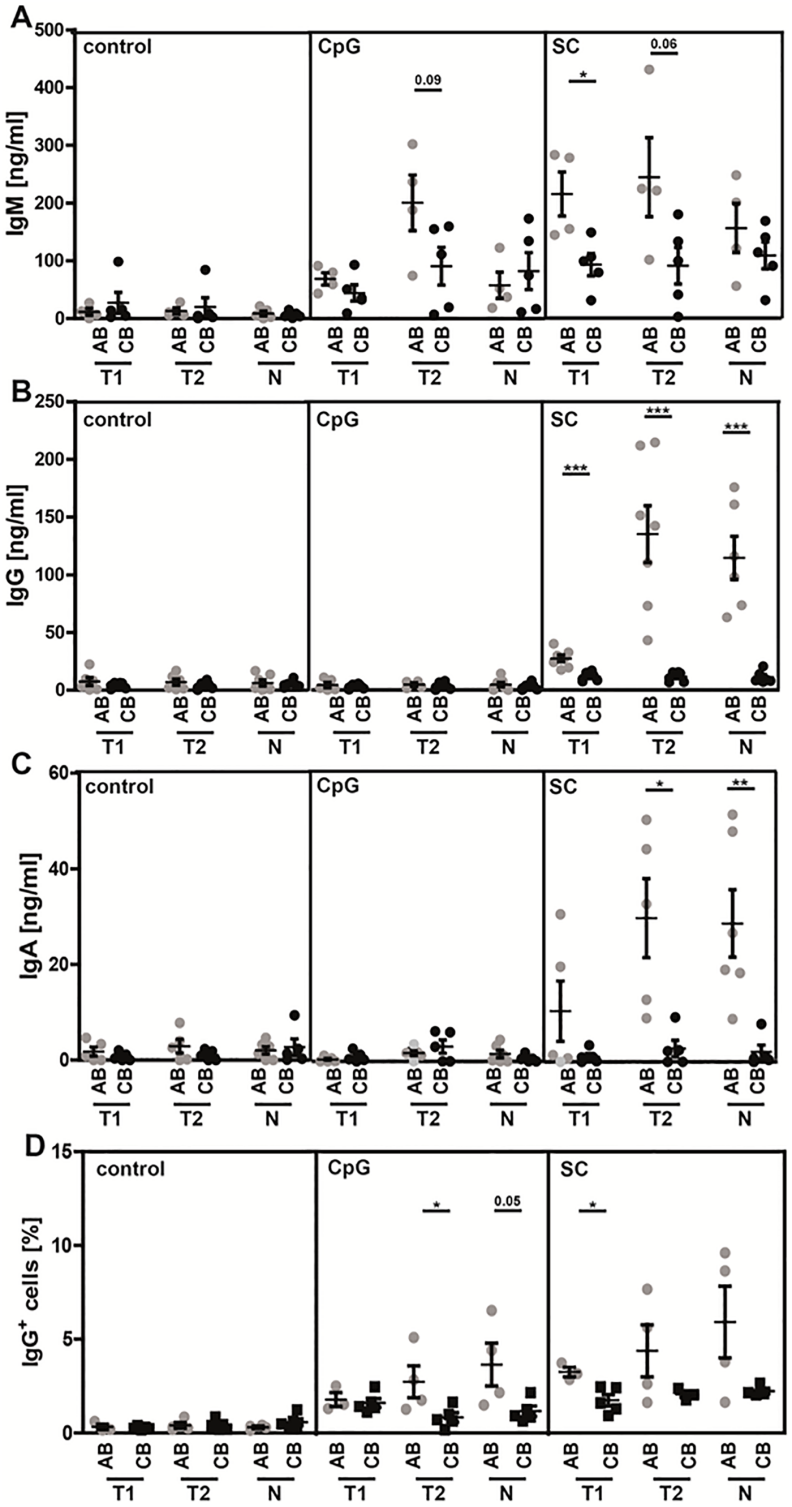
Impaired class-switching in neonatal B cells. Sort-purified adult and neonatal B cell subsets (transitional 1 & 2 B cells: T1 & T2; naïve mature B cells: N) were stimulated with CpG alone or in combination with IL-4, IL-21, anti-CD40, and BAFF (SC) for 8 days. After this time, cells were harvested and immunoglobulin levels for **(A)** IgM, **(B)** IgG, and **(C)** IgA, were determined in the supernatant by ELISA; (n = 5). **(D)** Surface IgG expression in sorted and stimulated (5 d; ctrl = not stimulated, with CpG or SC alone; AB: n≥3, CB: n = 5) B cells were determined by flow cytometry. Students *t*-test; * P<0.05, ** P<0.01, *** P<0.0001.

When stimulated with CpG, the highest amounts of IgM were produced by adult T2 B cells (Fig 4A). Stimulation with the SC resulted in significant higher IgM production of adult T1 B cells and a tendency towards significant higher IgM production of adult T2 B cells compared to their neonatal counterparts (T1: AB 214.8±75.8 ng/ml, CB 92.6±42.8 ng/ml; T2: AB 244.1±136.8 ng/ml, CB 90.8±70.8 ng/ml; p = 0.06).

Analysis of class-switched Ig revealed that in response to stimulation with CpG only small amounts of IgG or IgA were produced without any differences between adult and neonatal B cells. However, stimulation with the SC resulted in significantly increased IgG and IgA secretion by adult B cell subpopulations (IgG: T1: AB 27.3±7.7 ng/ml, CB 11.6±3.9 ng/ml; T2: AB 135.1±65.1 ng/ml, CB 11.5±4.2 ng/ml; Naïve: AB 114.5±45.8 ng/ml, CB 11.0±5.2 ng/ml); IgA: T1: AB 10.6±14.1 ng/ml, CB 1.1±1.4 ng/ml; p = 0.1724; T2: AB 30.0±18.5 ng/ml, CB 2.8±3.8 ng/ml; Naïve: AB 28.9±17.3 ng/ml, CB 2.1±3.3 ng/ml); (Fig 4B and 4C).

While IgM secretion of neonatal T1 and T2 B cells was only slightly, about 2fold decreased, IgG and IgA production was almost absent and over 10fold decreased in neonatal compared with adult B cell subsets. In order to test whether differences in IgG levels were directly due to alterations in CSR, we assessed surface expression of IgG on B cells after 5 days of stimulation. We found that CpG stimulation resulted in significantly higher IgG expression on T2 and naïve mature B cells, as well as in T1 B cells after stimulation with SC in adult B cells compared to neonatal B cells (CpG: T1: AB 1.8±0.6%, CB1.6±0.5%; T2: AB 2.7±1.7%, CB 0.8±0.5%; Naïve: AB 3.6±2.3%; CB 1.2±0.6%; SC: T1: AB 3.3±0.4%, CB 1.7±0.7%; T2: AB 4.4±2.8%, CB 2.1±0.3%; Naïve: AB 5.9±3.8%; CB 2.2±0.3%; Fig 4D). We therefore conclude that neonatal B cells have a severe defect in class-switching.

### Increased expression of miRNAs in neonatal B cells

So far, our data demonstrate key functional differences between neonatal and adult B cell subpopulations. Most importantly, neonatal B cells showed reduced class-switching capability. B cell differentiation and antibody responses are regulated by diverse cellular, environmental, but also epigenetic mechanisms [29, 40, 41]. In the course of these findings we next wanted to determine if epigenetic mechanisms contribute to the observed alterations. To evaluate this possibility we investigated miRNA expression.

**Fig 5:**
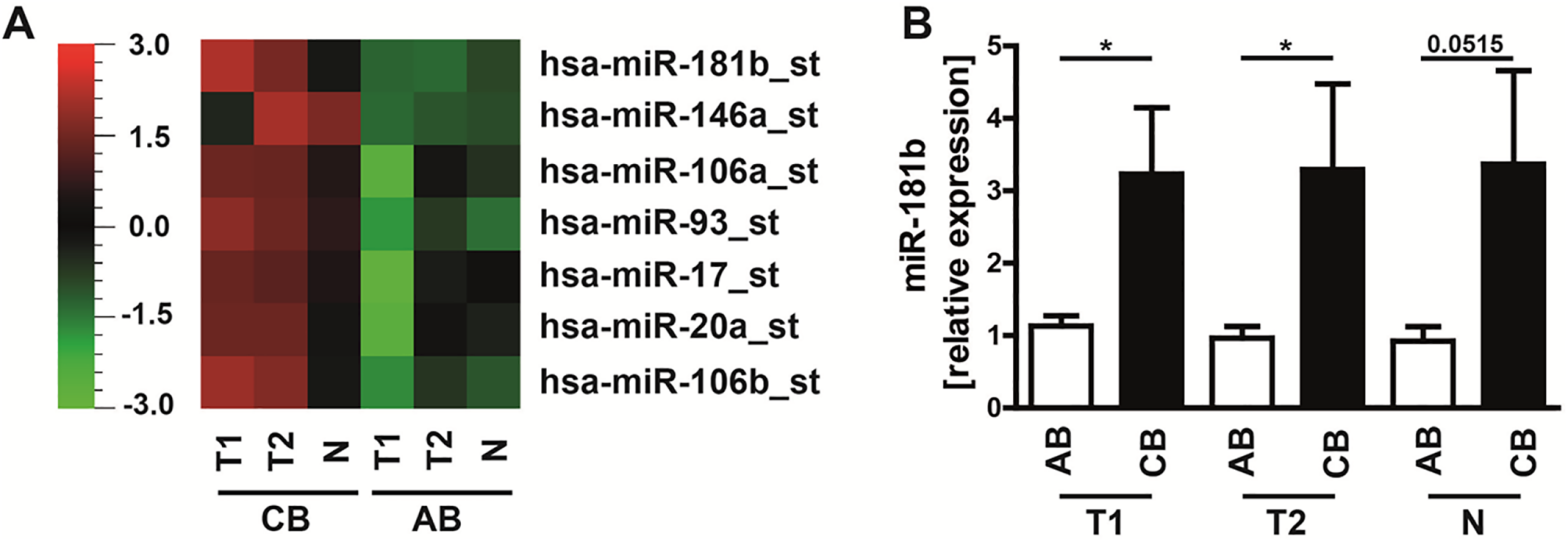
Significant differences in miRNA profiles of adult and neonatal B cells. **(A)** Analysis of miRNA expression of adult blood (AB) and neonatal cord blood (CB) (each n = 7; pooled) performed with QLUCORE analysis software and Multi Group Comparison of 1,105 human mature miRNAs; shown are differentially expressed miRNAs with a 3-fold difference between equivalent subsets; P<0.05. **(B)** QPCR analysis of miR-181b in B cell subpopulations (transitional 1 & 2 B cells: T1 & T2; naïve mature B cells: N) of neonatal and adult healthy donors; (n = 7); Students *t*-test; * P<0.05.

We therefore performed a screening of miRNA expression profiles of human neonatal and adult B cell subpopulations, using a miRNA microarray platform developed by Affymetrix, which analyzes 1,105 human mature miRNAs. Among differentially expressed miRNAs, we identified 7 miRNAs (miR-181b, miR-146a, miR-106a, miR-93, miR-17, miR-20a, and miR-106b) with more than 3-fold increased expression in B cells from neonates compared to adults (Fig 5A). Interestingly, no miRNA was expressed at a higher level in adult compared to neonatal B cells. The data obtained by microarray analysis were further validated by qPCR. We further analyzed miR-181b expression, which has been earlier described to impair CSR upon ectopic expression in activated B cells through negative regulation of activation-induced cytidine deaminase (AID) [42]. Quantitative PCR analysis confirmed significantly higher miR-181b expression in neonatal compared to adult B cell subpopulations (T1: AB 1.1±0.4, CB 3.2±2.4; p = 0.0433; T2: AB 1.0±0.5, CB 3.3 ±3.1, p = 0.0430; Naïve: AB 0.9±0.6; CB 3.4±3.4; p = 0.0515); (Fig 5B). No differences in expression levels could be observed between B cell subsets from either adult or neonatal cord blood.

### Deficiency of miR-181b results in increased CSR

Next, we investigated if elevated levels of miR-181b result in impaired CSR. Since transfection of primary human B cells is extremely difficult, we analyzed a previously established miR-181a/b-1 ko mouse model [34]. Due to the clustered genomic location of miR-181a-1 and miR-181b-1, both miRNAs are simultaneously knocked-out. However, we did not detect significant differences in the expression of miR-181a between neonatal and adult B cell subpopulations (data not shown). Hereupon, we hypothesize that possible differences in CSR are based on the knock-out of the miR-181b gene.

First, we performed a careful flow cytometric analysis of splenic B cells determining T1, T2, follicular mature (FM), marginal zone precursor (MZp) and marginal zone (MZ) B cells in adult and neonatal miR-181a/b^+/-^ (het) and miR-181a/b^-/-^ (ko) mice as previously described [19] (S9 Fig and Table 2). No significant differences in the composition of B cell subpopulations were found between miR-181a/b^+/-^ and miR-181a/b^-/-^ mice.

**Table 2:** Frequency of B cell subsets in miR-181a/b het and ko mice.

	Adult		Neonatal	
Subset	het	ko	het	ko
**Splenocytes (absolute counts)**	8.9 x 10^7^ ± 5.0 x 10^7^	1.1 x 10^8^ ± 5.0 x 10^7^	5.1 x 10^6^ ± 4.3 x 10^6^	3.6 x 10^6^ ± 1.9 x 10^6^
• **B cells**	40.2 ± 2.3	37.6 ± 5.2	21.9 ± 7.6	24.6 ± 15.5
– **T1**	24.8 ± 11.7	28.1 ± 9.6	96.2 ± 2.5	96.6 ± 0.8
– **T2**	2.4 ± 1.3	3.0 ± 1.5	0.8 ± 0.6	0.7 ± 0.6
– **MZp/MZ**	12.1 ± 3.0	14.3 ± 4.5	0.6 ± 0.8	0.1 ± 0.1
– **MZp**	23.7 ± 3.7	22.0 ± 5.6	1.5 ± 2.5	2.6 ± 3.2
– **MZ**	75.5 ± 4.0	77.1 ± 6.0	64.5 ± 55.9	53.8 ± 55.3
– **FM**	58.1 ± 11.5	51.1 ± 9.0	0.3 ± 0.3	0.5 ± 0.2

FM: follicular mature; MZ: marginal zone; MZp: marginal zone precursor; T1 & T2: transitional 1 & 2

Adult: het/ko n = 11 each; Neonatal: het/ko n18/14 (pooled)

The frequency of total B cells (as percentage of total spleen cells; splenocytes), T1 & T2, MZp/MZ, and FM cells (as percentage of total B cells) as well as MZ and MZp cells (as percentage of MZp/MZ cells) is shown.

**Fig 6:**
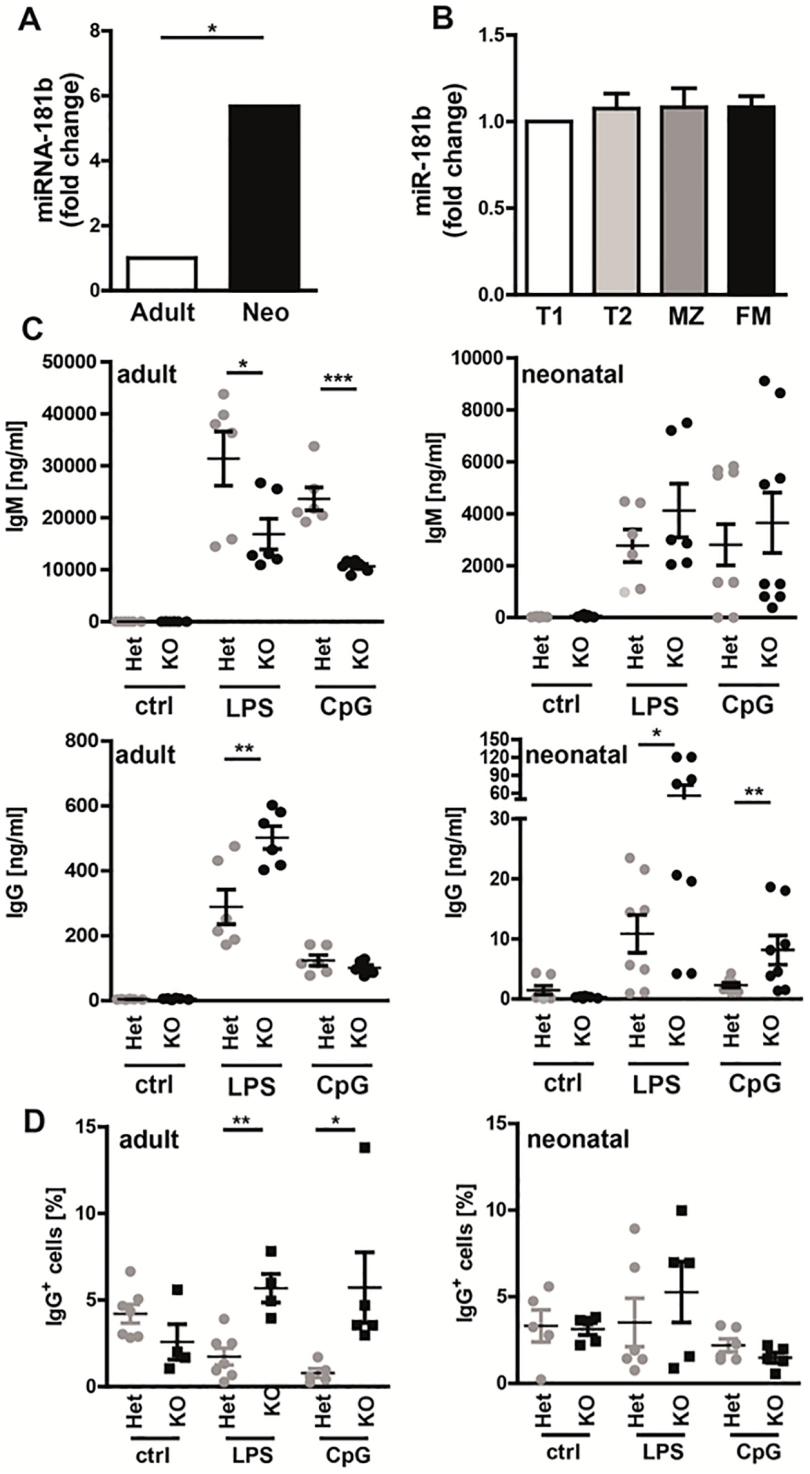
MiR-181b plays a crucial role in the regulation of class-switching to IgG. **(A, B)** QPCR analysis for mature miR-181b expression in **(A)** total splenocytes of adult and neonatal miR-181a/b^+/-^ het mice (adult: n = 11, neo: n = 4 (pooled from 18 mice)) and **(B)** sorted B cell subpopulations (transitional 1 &2 (T1 & T2), marginal zone (MZ), and follicular mature (FM); n = 5) of adult C57BL/6J mice. Relative expression was calculated by using the standard curve method. **(C)** IgM and IgG levels in supernatants from cultured and stimulated (5 d; ctrl = not stimulated, with LPS or CpG alone; adult n = 6, neonatal: n≥6) adult and neonatal splenic cells isolated from miR-181a/b^+/-^ and miR-181a/b^-/-^ mice were determined by ELISA. **(D)** Surface IgG expression in stimulated (5 d; ctrl = not stimulated, with LPS or CpG alone; adult n = 6; neonatal: n = 51(Het)/34(KO), pooled to n≥5(Het)/4(KO)) adult and neonatal splenic cells isolated from miR-181a/b^+/-^ and miR-181a/b^-/-^ mice were determined by flow cytometry. Students *t*-test; * P<0.05, ** P<0.01, *** P<0.001.

We subsequently determined miR-181b expression. Similar to the human data, miR-181b expression was significantly up-regulated in neonatal compared to adult miR-181a/b^+/-^ splenic B cells (Fig 6A). No difference in miR-181b expression was found between splenic B cell subpopulations of adult mice (Fig 6B).

Next, Ig production after *in vitro* stimulation with the TLR4 ligand LPS or CpG of isolated splenic B cells was analyzed. Our data show that in adult mice, miR-181a/b^+/-^ B cells produced significant larger amounts of IgM, but lower amounts of IgG compared to miR-181a/b^-/-^ B cells (IgM: LPS: het 31396.0±12807.4 ng/ml, ko 16853.9±7242.2 ng/ml; CpG: het 23637.9±5406.2 ng/ml, ko 10663.6±1122.5 ng/ml; IgG: het 289.1±131.2 ng/ml, ko 502.5±85.5 ng/ml). In neonatal mice, no significant differences were found in respect to IgM production. However, the production of IgG was significantly increased after stimulation with both LPS and CpG in miR-181a/b^-/-^ compared with miR-181a/b^+/-^ B cells (Fig 6C; LPS: het 10.9.9±8.9 ng/ml, ko 55.9±49.7 ng/ml; CpG: het 2.6±1.1 ng/ml, ko 8.2±6.1 ng/ml). Similar to the analysis of human samples we determined IgG expression on splenic cells after stimulation for 5 days with CpG and LPS to confirm class-switching. The data show that significantly more adult miR-181a/b^-/-^ splenic cells are positive for IgG compared to splenic cells from miR181a/b^+/-^ mice after stimulation with LPS or CpG. (LPS: Adult: het 2.0±2.1%, ko 5.7±4.6%; Neonatal: het 3.5±3.4%, ko 5.3±3.9%; CpG: Adult: het 1.7±1.3%, ko 5.7±1.7%; Neonatal: het 2.2±0.9%, ko 1.5±0.6%; Fig 6D). In neonatal spleens the data only show a tendency towards a higher percentage of IgG^+^ B cells in miR-181a/b^-/-^ compared to miR-181a/b^+/-^ mice when stimulated with LPS.

Consequently, the deletion of miR-181a/b led to a significant increase in CSR and production of IgG in both adult and neonatal murine splenic B cells when stimulated via TLR-4 with LPS. This effect was also seen in neonatal B cells but not adult B cells when stimulated with CpG.

### Enhanced expression of activation-induced cytidine deaminase (AID; *Aicda*) in activated neonatal B cells

**Fig 7:**
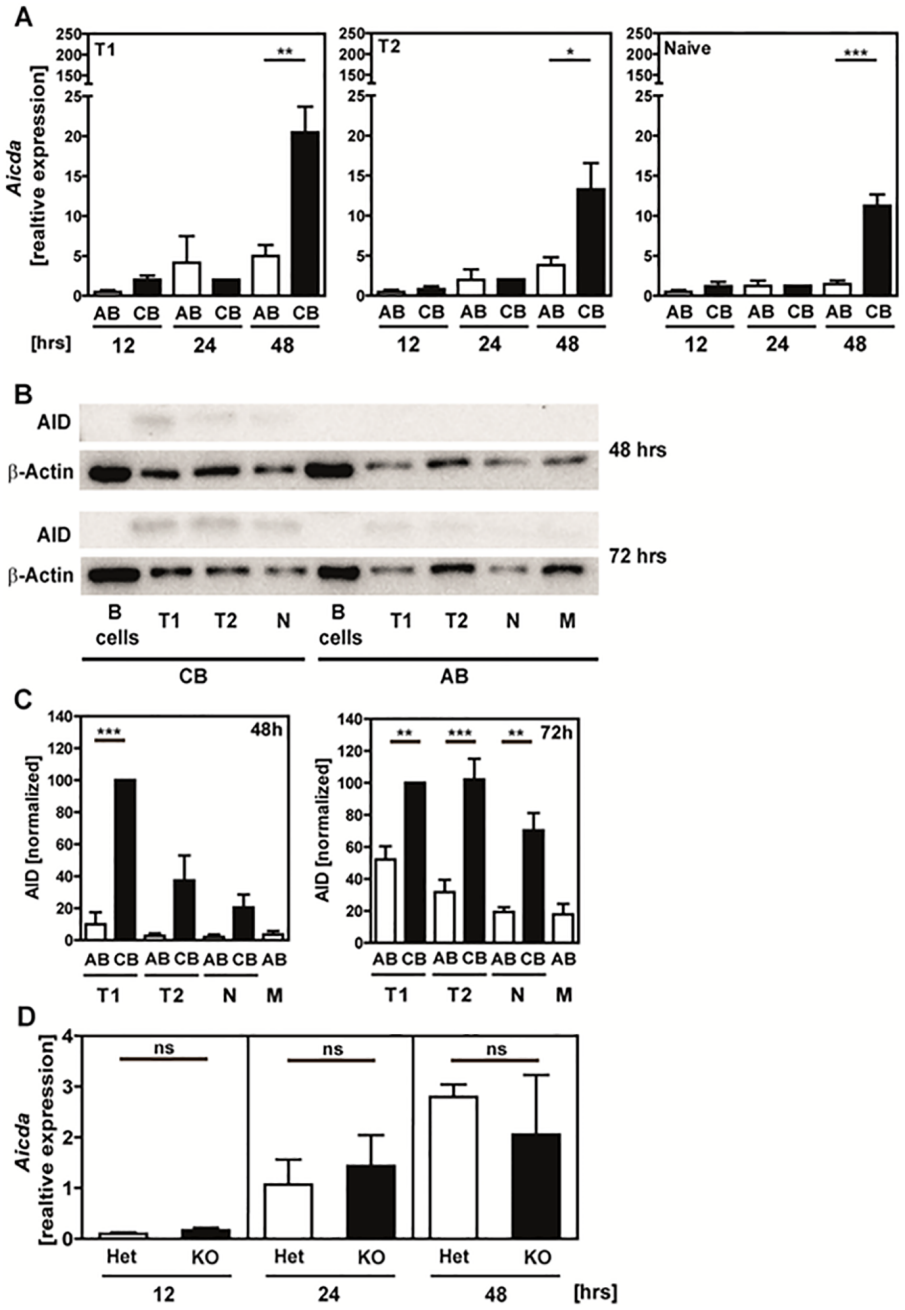
High expression of *Aicda* mRNA and AID protein in stimulated neonatal B cells. **(A)** QPCR analysis for *Aicda* transcripts in human B cells cultured for 12, 24, and 48h with IL4+ IL-21+antiCD40+CpG+BAFF. Expression is normalized to GAPDH and is calculated relative to expression in not stimulated control B cells at each time point. Mean and SEM of n ≥ 3 samples are shown. **(B)** AID protein expression after 48h and 72h of stimulation in sorted adult blood (AB) and neonatal cord blood (CB) B cell subpopulations analyzed by Western blot; β-Actin was used as housekeeping protein; 4 μg protein/sample was applied. One representative run is shown. **(C)** Normalized results of n ≥ 4 independent experiments. **(D)** Quantitative RT-PCR analysis for *Aicda* transcripts in adult murine spleen cells cultured and stimulated for 12, 24, and 48h with LPS+CpG+anti-CD40+IL-4. Expression is normalized to GAPDH and calculated relative to expression in not stimulated spleen cells. Mean and SEM of n ≥ 3 samples are shown. Statistical analysis was performed using for **(A)** Student´s *t* test and for **(C)** 1way ANOVA. * P < 0.05, ** P < 0.01. *** P < 0.001.

It has been previously suggested that miR-181b negatively regulates *Aicda* in B cells [42]: Ectopic expression of miR-181b in activated B cells resulted in down-regulation of *Aicda* mRNA and AID protein levels thereby impairing CSR. We therefore determined *Aicda* mRNA and AID protein expression. Our results show higher *Aicda* expression levels in all three analyzed human neonatal B cell subsets after stimulation with the stimulation cocktail for 48h compared to their adult counterparts (T1: AB 5.0±2.7, CB 20.5±6.4; T2: AB 3.8±2.0, CB 13.3±5.7; T1: AB 1.5±0.9, CB 11.3±2.8; Fig 7A). Similarly, neonatal B cells showed generally higher AID protein levels determined 72h after stimulation in comparison to their adult counterparts, with highest levels in T1 B cells (Fig 7B and 7C). No differences in *Aicda* expression were found in stimulated B cells from miR-181a/b^-/-^ and miR-181a/b^+/-^ mice (Fig 7D). Thus, in contrast to the above-mentioned publication by de Yebenes [42], high miR-181b levels were not associated with down-regulation of *Aicda* or AID in our study.

## Discussion

The immaturity of the neonatal immune system is a result of both qualitative and quantitative differences between neonatal and adult immune cells. In respect to the B cell compartment several studies have demonstrated an altered composition of B cell subpopulations [16, 18] in addition to functional differences [7, 8, 24, 25]. However, because of their different subset composition, comparisons between adult and neonatal total B cells might give misleading information. In the current study we therefore directly compared corresponding human B cell subpopulations from neonatal CB and peripheral AB, namely T1, T2 and naïve mature B cells gated as previously described [43–45]. Notably, we did not separate and analyze T3 B cells which can be separated from CD24^int^CD38^int^ naïve mature B cells by differential expression of the ABCB1 transporter [46]. In cord blood, a higher proportion is contained within CD24^int^CD38^int^ naïve mature B cells compared to adult peripheral blood. However, no differences in calcium flux or proliferation between naïve mature and T3 B cells have been observed and other functional data including Ig production have not been described so far. Therefore, we did not include this subset in our analysis.

Our data demonstrate similar phenotypical characteristics, but significant functional differences between neonatal and adult B cell subsets, in particular a severe defect in CSR of neonatal B cells. Further analysis revealed up-regulation of miR-181b which has been previously described to be involved in CSR [42]. Consistent with this idea stimulated B cells from miR-181a/b deficient mice produced significantly more IgG than B cells from miR-181a/b heterozygous mice.

A variety of surface molecules were previously shown to be differentially expressed on neonatal compared to adult B cells namely CD62L, CCR7 [47], CD22 [20], CD80, CD86/CD40 [14] and BAFF receptors [48–50].

In contrast to these publications, we could not detect significant differences in surface marker expression. This obvious controversy can be explained by the fact that we analyzed corresponding B cell subpopulations, whereas previous studies always characterized bulk B cells from murine spleen or human blood. The only detectable difference in surface expression that was maintained at the level of B cell subpopulations was IgM as previously reported [23]. The high expression of IgM in neonatal B cells most likely accounts for the observed enhanced calcium flux (Ca^2+^-flux) and phosphorylation of tyrosine residues in response to anti-IgM stimulation. We therefore propose that early BCR signal transduction is unaffected in neonatal B cells, but that overall BCR signaling strength per cell is enhanced due to increased IgM expression. Stronger BCR signals can promote cell death as shown in transitional B cells [51] and leukemia cell lines [52]. When we stimulated neonatal B cell subpopulations via the BCR in addition to TLR, both survival and proliferation were significantly reduced compared to adult B cells, although neonatal B cells are generally able to proliferate as much as adult B cells. They even better proliferate in response to TLR9 ligation despite equal TLR9 expression. Consistently, previous publications demonstrate diverse results in respect to proliferative responses of neonatal B cells depending on the stimulus [14, 20]. In contrast to the varying proliferative responses, the evidence for defective CSR in neonatal B cells is much more consistent throughout the literature. In response to different stimuli including pokeweed mitogen (PWM) in the presence of CD4^+^ T cells [53], CpG [5], or CD40L alone or in addition with IL-10 [14, 15], neonatal B cells secreted significantly lower levels of IgG and IgA compared to adult B cells. Because most immunoglobulins are produced by CD27^+^ memory B cells [54], a recent study compared Ig production of CD27^-^ naïve B cells from newborns and adults [55]. In response to combined TLR, CD40 and IL-2 stimulation, the percentages of IgG secreting cells were significantly lower for newborn naïve B cells relative to adult counterparts. Based on our data we conclude that the inability of neonatal B cells to perform CSR results from a B cell intrinsic defect. Notably, we already see slightly lower production of IgM in neonatal compared to adult B cells, although this is not as severe as for IgG and IgA production. There is a possibility that this results from delayed IgM secretion or an overall impairment of Ig production [56].

We hypothesized that epigenetic modulations contribute to the observed effect because epigenetics are involved in early live programming and postnatal development [57–59].

In our analysis, only 7 out of 1105 human mature miRNA were more than 3-fold up-regulated in neonatal compared to adult sort-purified B cell subpopulations, whereas no miRNA was found to be expressed at a lower level. Amongst these are five miRNAs (miR-17, miR-20a, miR-93, miR106a, and miR-106b) which belong to the three highly conserved polycistronic, paralogue clusters miR17~92, miR106b~25, and miR106a~363 [60–62], which originated via a series of duplication and deletion events during early vertebrate evolution and which functionally cooperate in regulating embryonic development [63].

Many studies, mostly in the murine system, have been performed to analyze these clusters, the interplay between them, and their effects on B cells. They revealed that the miR-17~92 cluster is highly expressed in embryonic cells with an important role in fetal and adult B cell development, as well as an important role in the regulation of B cell survival [61]. Absence of miR17~92 results in impaired B-cell development at the pro- to pre-B cell transition with Bim as key functional target, and reduced numbers of pre-B cells [64]. Jiang et al. [65] furthermore stated an important role in B-cell proliferation, protection of B cells from death, support of IFNγ production and suppressing T cell differentiation by the miR17~92 cluster. Herein direct target of miR-17 and miR-20a is TGFβRII, in that they are involved in TGF-β signaling [66].

Moreover, miR-146a was found to be expressed at a higher level in neonatal B cell subsets. This miRNA was shown to have multiple targets, including IRAK1, IRAK2, TRAF6, IRF-5, FAS and FADD. Many studies suggest a role for miR-146a in cell apoptosis and cytokine secretion [67] but it also seems so play a role in B cell development based on functional defects in murine miR-146a deficient B cells [68]. Other studies have also shown higher miR-146a expression in different neonatal compared to adult cells, namely murine intestinal epithelial cells [69], human granulocytes and T cells [70].

In addition to elevated levels of miR-181a, neonatal human T cells also express higher levels of miR-181b [71]. While we did not find significant differences in miR-181a expression in B cells, this could point to a T cell-specific alteration. Furthermore, significantly elevated serum levels of miR-181b were detected in younger compared to older mice [72]. In recent studies, elevated levels of miR-181b were shown to impair CSR either in transduced mouse B cells, in the Ramos Burkitt lymphoma cell line or B cells treated with the histone deacetylase inhibitors valproic acid and butyrate [42, 73]. Based on these reports, our data suggest that elevated expression of miR-181b is at least partially responsible for defective CSR in neonatal B cell subsets. We could confirm this assumption by analyzing miR-181b deficient mice: deficiency of miR-181b increased CSR to IgG of *in vitro* stimulated B cells. The effect was much more pronounced in neonatal compared to adult B cells. This is probably due to the fact that miR-181b is already expressed at a lower level in adult B cells, and therefore a complete deletion only results in a graduated smaller decrease. Because of the low number of splenocytes in neonatal mice, we were not able to analyze sort-purified B cell subsets and subsequently determined Ig production in total splenocytes. Since the neonatal B cell compartment mainly consists of transitional B cells (Table 2), lower amounts of IgG are secreted compared to adult splenic cells as shown in Fig 6C. Notably, miR-181a/b^-/-^ B cells from adult, but not neonatal mice produced lower amounts of IgM compared to miR-181a/b^+/-^ mice. This could be the results of more B cells undergoing class-switching, thereby remaining fewer B cells secreting IgM.

MiR-181b is known to regulate the expression of multiple genes thereby influencing many different processes. DeYebenes and White [42, 73] suggested an important role for miR-181b in regulating *Aicda*, which seems to be negatively regulated in B cells, influencing this way the CSR process. However, in contrast to this publication [42, 73], elevated miR-181b levels were not associated with decreased expression of *Aicda* and AID in our study. This discrepancy might be explained by differences in experimental conditions. Whereas prior studies employed ectopic expression of AID in *in vitro* cultured B cells, we analyzed *Aicda* expression directly *ex vivo* comparing either adult and neonatal states or miR-181a/b-deficient and sufficient scenarios. Interestingly, a recent study on miR-17~92 targets during early B cell development showed that opposing changes in miRNA expression levels resulted in the identification of fundamentally distinct groups of miRNA targets [74].

Nevertheless, it remains unresolved why high AID levels in neonatal B cells are not sufficient for proper CSR. Possibly, other molecules involved in CSR downstream of AID, could be functionally affected. For example, deficiency of UNG or Msh6 results in primary human immunodeficiency presenting as a class-switching defect, also called Hyper-IgM syndrome [75]. Alternatively, additional factors like membrane-associated proteins [76] or other epigenetic factors can affect CSR. Other direct targets or indirect interactions of miR-181b, which could be involved in CSR include TLR4 [77], TGF-beta and SMAD signaling [78, 79], PI3K and MAPK pathways [80], Bim [81], STAT3 [82], PDCD10 and GATA6 [83]. So far many of these targets have been analyzed in specific cell types and their role in lymphocytes still has to be investigated.

With this, our data provide new detailed insight into the mechanisms contributing to the immaturity of the neonatal B cell compartment and highlight within a physiological setting the importance of differentially regulated miRNA. Diminished immune responses and antibody production might be reasonable during postnatal adaptation where newborns encounter many new antigens, and overwhelming immune responses could harm the new individual. However, in respect to vaccination in early live, it is desirable to improve the generation of high-affinity and class-switched antibodies. Based on our data, this could be achieved by designing adjuvants that downregulate miR-181b. Therapeutic modification of miRNA expression might therefore represent a promising target in the future.

## Supporting information

S1 Fig Antibodies and stimulation reagents used in each method. (TIF)

S2 Fig Antibody panels for immunophenotypical B cell subset characterization. (TIF)

S3 Fig Primer sequences. (TIF)

S4 Fig Different composition of the adult and neonatal B cell compartment. Gating strategy for flow cytometric analysis of B cell subpopulations in adult blood (AB) and neonatal cord blood (CB). Lymphocytes were stained for the surface markers CD19, CD24, CD27 and CD38 and pre-gated for discrimination between transitional 1 & 2 (T1 & T2) and naive mature B cells (CD19^+^CD27^-^) and memory B cells (CD19^+^CD27^+^). CD19^+^CD27^-^ B cells were subsequently separated into T1 (CD24^++^CD38^++^), T2 (CD24^+^CD38^+^) and naive mature B cells (CD24^-^CD38^-^). Shown is one representative example for each AB and CB; displayed are percentages of CD19^+^ B cells (left panel) and CD19^+^CD27^-^ B cells (right panel).
(TIF)

S5 Fig Confirmation of immaturity of sort-purified B cell subpopulations by staining for IgM and IgD. To confirm that previously sorted T1, T2, and naïve mature B cells are purely immature and not contaminated by memory B cells, the sorted subsets were stained for surface IgM and IgD and subsequently analyzed by flow cytometry.
(TIF)

S6 Fig Ca^2+^-Flux analysis of all experiment performed. Isolated adult and neonatal B cells were surface-stained for B cell subset discrimination (transitional 1 & 2 B cells: T1 & T2; naïve mature B cells: N) and stimulated via the BCR for flow cytometric determination of Ca^2+^-Flux by calculating the Indo-1 ratio measured for 5 min.
(TIF)

S7 Fig Phosflow analysis of all experiments performed. Isolated adult and neonatal B cells were surface-stained for B cell subset discrimination (transitional 1 & 2 B cells: T1 & T2; naïve mature B cells: N) and stimulated via the BCR for flow cytometric determination of the pTyr status at 1, 2, 5, and 10 min.
(TIF)

S8 Fig Activated adult and neonatal B cell subpopulations show no significant differences in survival. Activated B cell subpopulations were analyzed by flow cytometry for cell survival by gating on forward-sideward scatter: **(A)** in sorted human adult B cell subsets over time (0h, 18h, 30h, and 54h; n = 3) after stimulation with either CpG or stimulation cocktail (SC); **(B)** in sorted human adult (n = 4) and neonatal B cell subsets (n = 5) after 5d stimulation with either medium control, CpG, or SC; **(C)** in splenocytes of adult and neonatal miR181a/b Het (adult n = 6; neonatal n = 51, pooled in ≥5 samples) and KO (adult n = 6; neonatal n = 34, pooled in 4 samples) mice after 5d stimulation with either medium control, CpG, LPS, or SC.
(TIF)

S9 Fig Different composition of the adult and neonatal B cell compartment in mice. Gating strategy for flow cytometric analysis of B cell subpopulations in splenic cells of adult and neonatal miR-181a/b^+/-^ mice. Spleen cells were stained for CD19, CD21, CD23 and CD24 and gated for discrimination between marginal zone precursor/marginal zone (MZp/MZ; CD21^++^CD24^++^), follicular mature (FM; CD21^int/low^CD24^int^), and transitional 1 and 2 (T1: CD21^int/low^CD24^++^, T2: CD21^int^CD24^++^) B cells. MZp/MZ B cells were subsequently gated for MZ (CD21^+^CD23^-^), and MZp B cells (CD21^+^CD23^+^). Shown is one representative example for adult and neonatal mice; displayed are percentages of CD19^+^ B cells (left panel: adult and right panel: neonates), and MZp/MZ B cells (middle panel: adult).
(TIF)
