# Supplementary figures and images for: Decreased production of class-switched antibodies in neonatal B cells is associated with increased expression of miR-181b
Source: PLoS One. 2018 Feb 1;13(2):e0192230. doi: 10.1371/journal.pone.0192230 (PMC5794184; doi:10.1371/journal.pone.0192230)

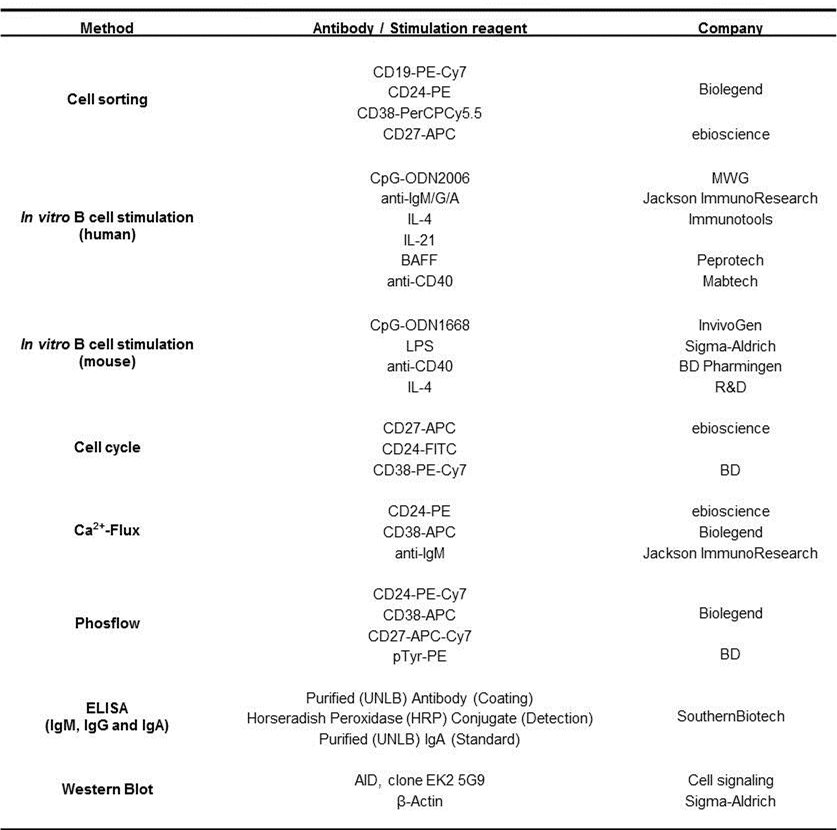

Supplement: S1 Fig — (TIF) [file pone.0192230.s001.tif]

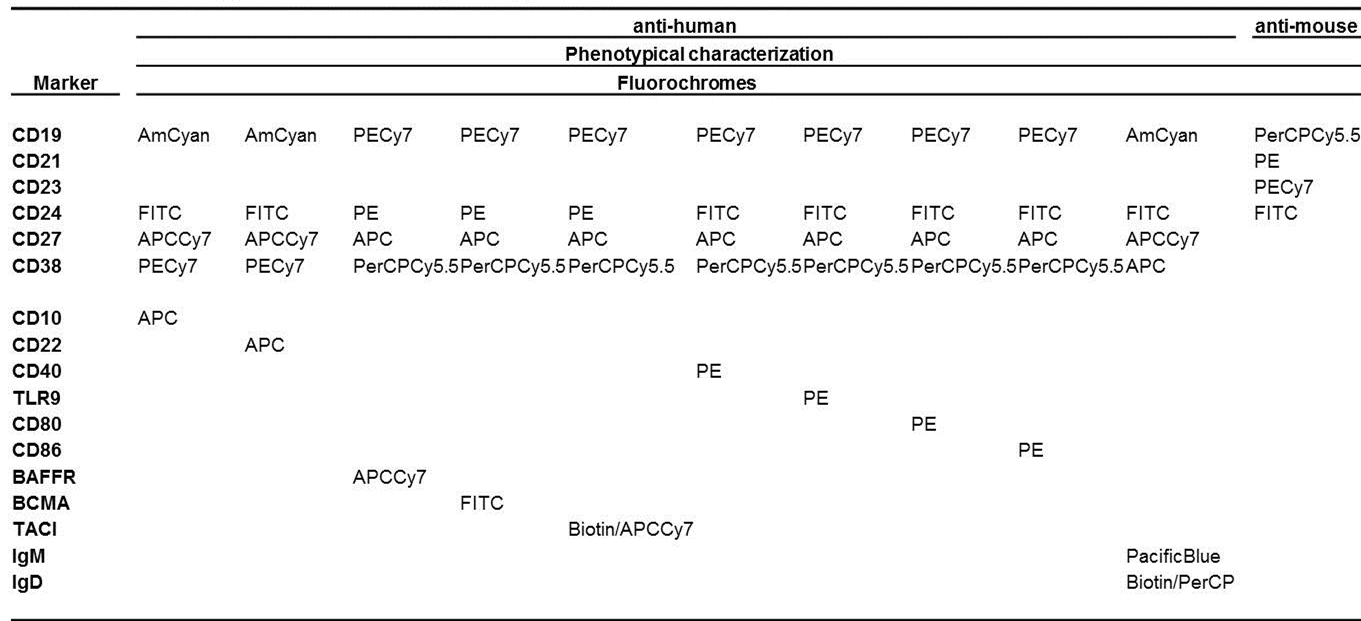

Supplement: S2 Fig — (TIF) [file pone.0192230.s002.tif]

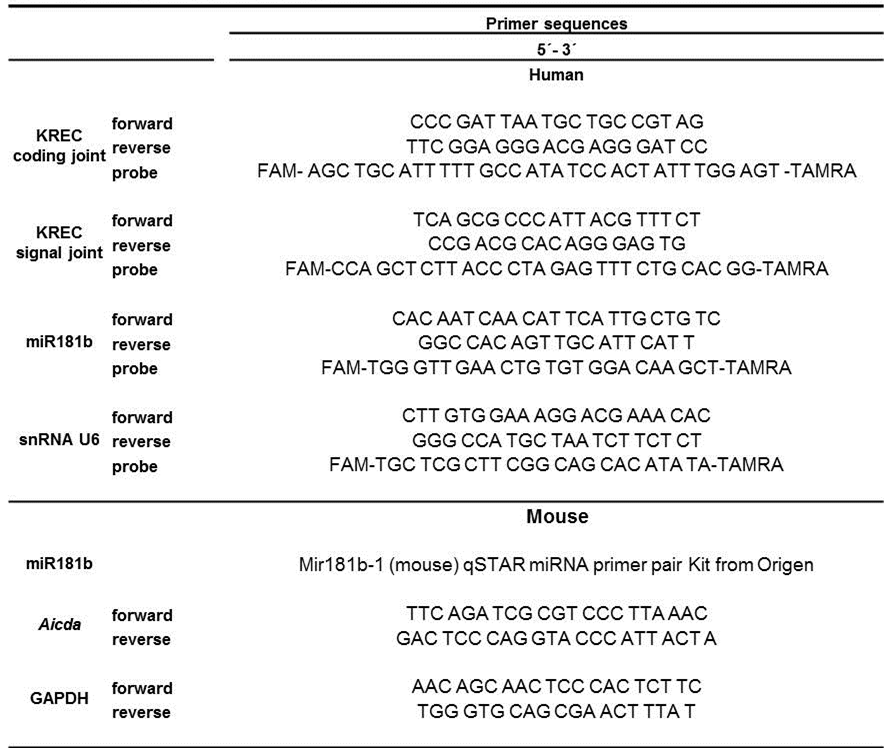

Supplement: S3 Fig — (TIF) [file pone.0192230.s003.tif]

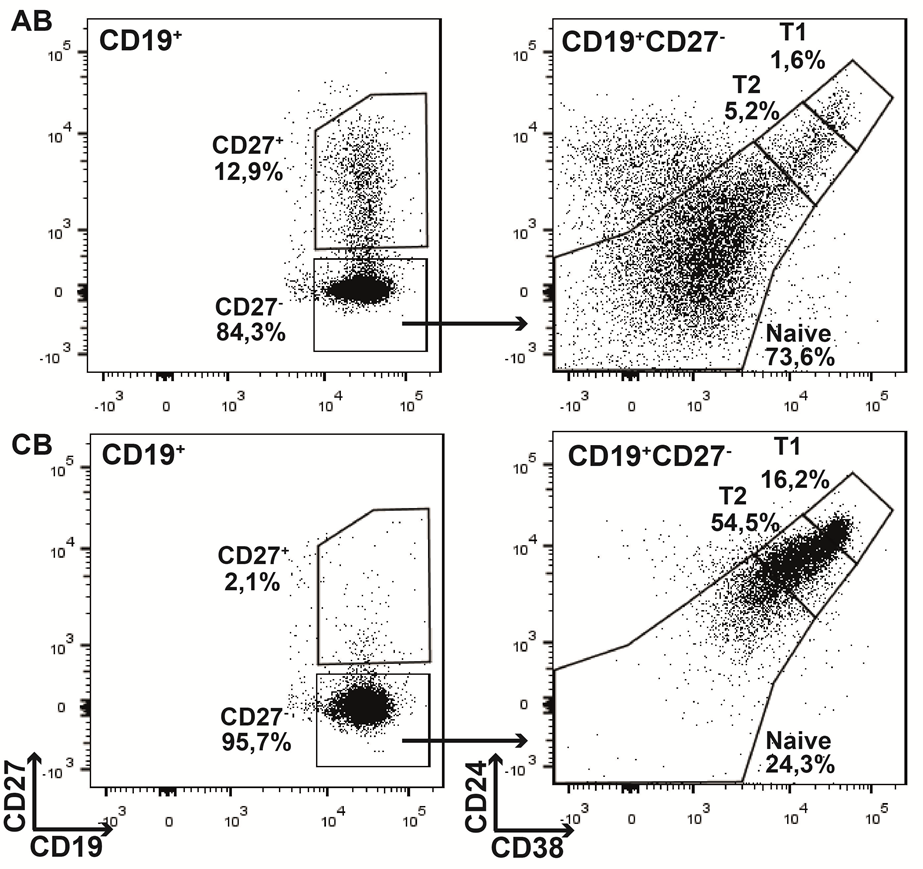

Supplement: S4 Fig — Gating strategy for flow cytometric analysis of B cell subpopulations in adult blood (AB) and neonatal cord blood (CB). Lymphocytes were stained for the surface markers CD19, CD24, CD27 and CD38 and pre-gated for discrimination between transitional 1 & 2 (T1 & T2) and naive mature B cells (CD19+CD27-) and memory B cells (CD19+CD27+). CD19+CD27- B cells were subsequently separated into T1 (CD24++CD38++), T2 (CD24+CD38+) and naive mature B cells (CD24-CD38-). Shown is one representative example for each AB and CB; displayed are percentages of CD19+ B cells (left panel) and CD19+CD27- B cells (right panel). (TIF) [file pone.0192230.s004.tif]

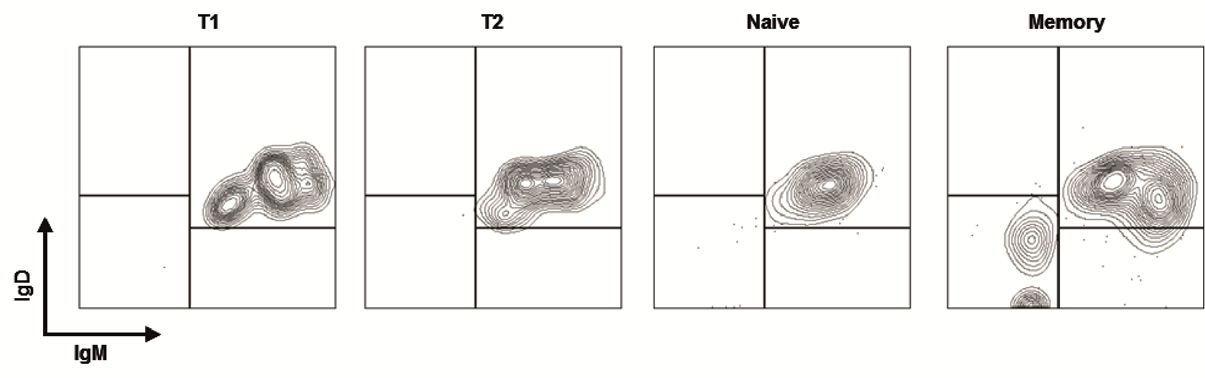

Supplement: S5 Fig — To confirm that previously sorted T1, T2, and naïve mature B cells are purely immature and not contaminated by memory B cells, the sorted subsets were stained for surface IgM and IgD and subsequently analyzed by flow cytometry. (TIF) [file pone.0192230.s005.tif]

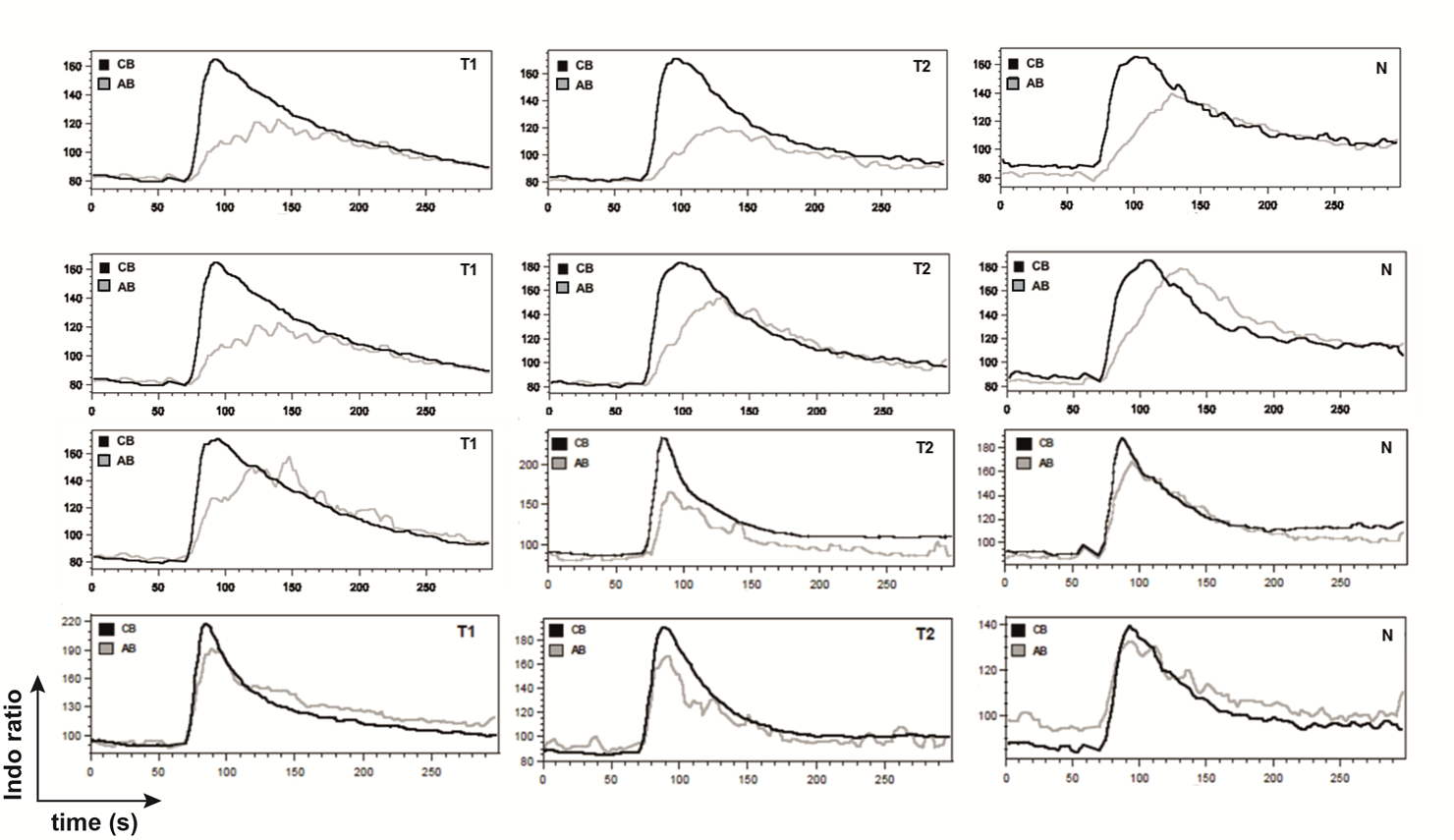

Supplement: S6 Fig — Isolated adult and neonatal B cells were surface-stained for B cell subset discrimination (transitional 1 & 2 B cells: T1 & T2; naïve mature B cells: N) and stimulated via the BCR for flow cytometric determination of Ca2+-Flux by calculating the Indo-1 ratio measured for 5 min. (TIF) [file pone.0192230.s006.tif]

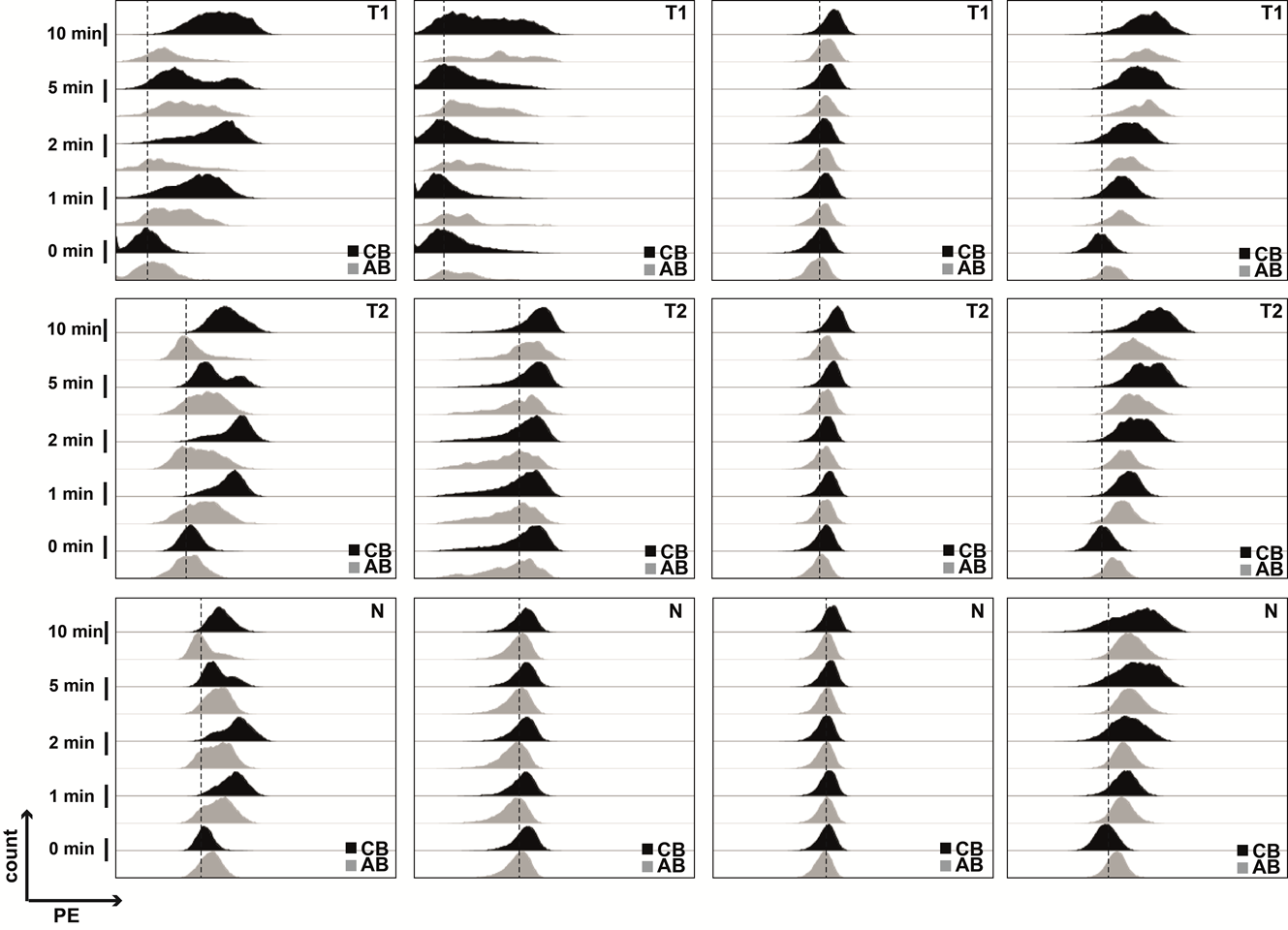

Supplement: S7 Fig — Isolated adult and neonatal B cells were surface-stained for B cell subset discrimination (transitional 1 & 2 B cells: T1 & T2; naïve mature B cells: N) and stimulated via the BCR for flow cytometric determination of the pTyr status at 1, 2, 5, and 10 min. (TIF) [file pone.0192230.s007.tif]

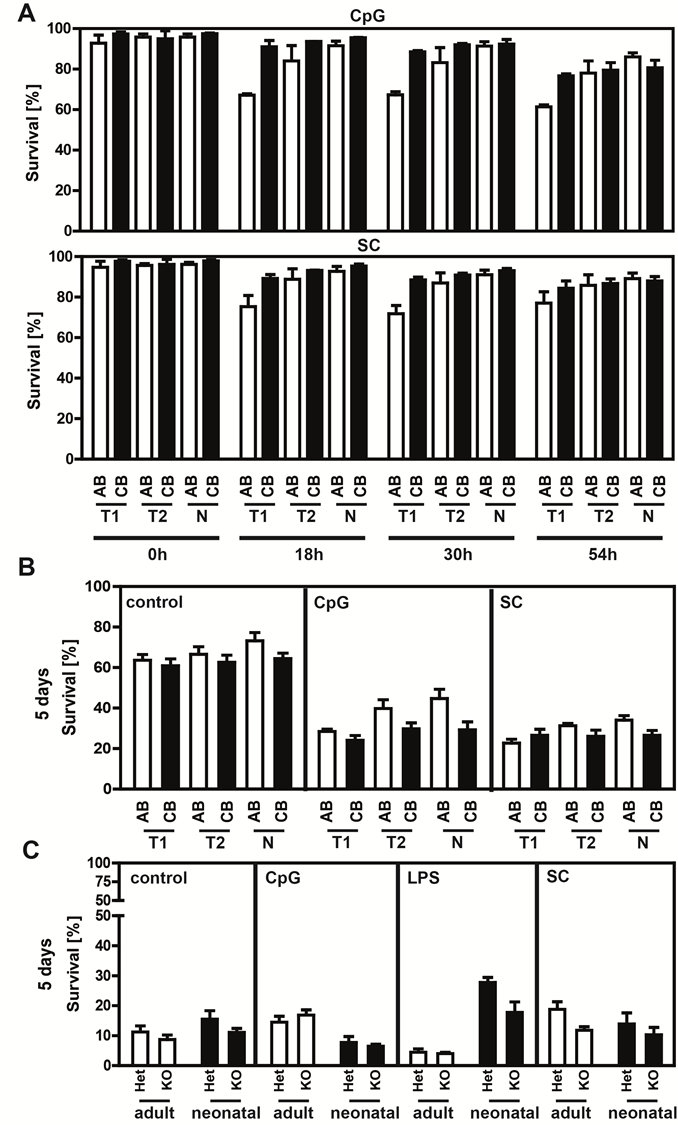

Supplement: S8 Fig — Activated B cell subpopulations were analyzed by flow cytometry for cell survival by gating on forward-sideward scatter: (A) in sorted human adult B cell subsets over time (0h, 18h, 30h, and 54h; n = 3) after stimulation with either CpG or stimulation cocktail (SC); (B) in sorted human adult (n = 4) and neonatal B cell subsets (n = 5) after 5d stimulation with either medium control, CpG, or SC; (C) in splenocytes of adult and neonatal miR181a/b Het (adult n = 6; neonatal n = 51, pooled in ≥5 samples) and KO (adult n = 6; neonatal n = 34, pooled in 4 samples) mice after 5d stimulation with either medium control, CpG, LPS, or SC. (TIF) [file pone.0192230.s008.tif]

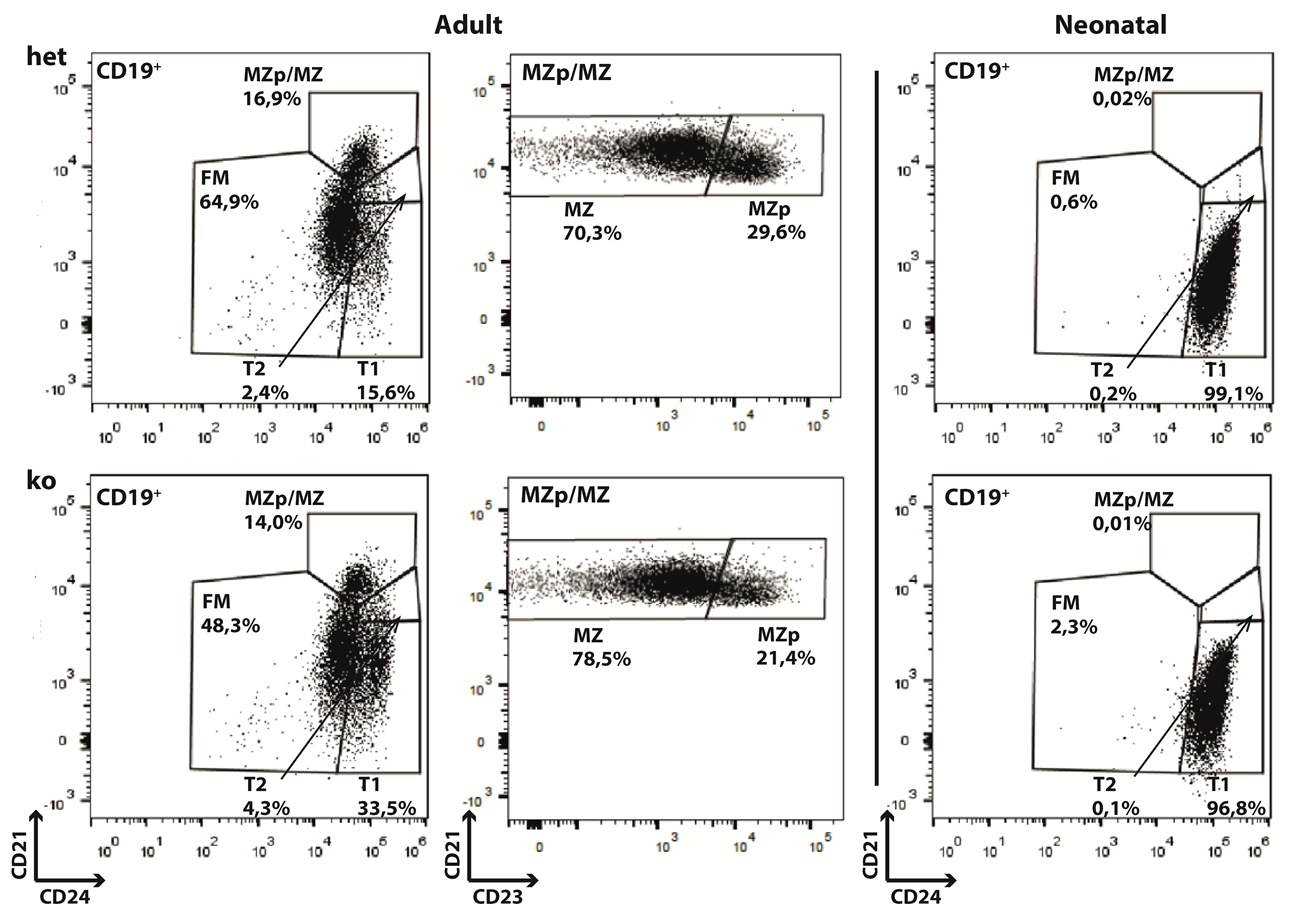

Supplement: S9 Fig — Gating strategy for flow cytometric analysis of B cell subpopulations in splenic cells of adult and neonatal miR-181a/b+/- mice. Spleen cells were stained for CD19, CD21, CD23 and CD24 and gated for discrimination between marginal zone precursor/marginal zone (MZp/MZ; CD21++CD24++), follicular mature (FM; CD21int/lowCD24int), and transitional 1 and 2 (T1: CD21int/lowCD24++, T2: CD21intCD24++) B cells. MZp/MZ B cells were subsequently gated for MZ (CD21+CD23-), and MZp B cells (CD21+CD23+). Shown is one representative example for adult and neonatal mice; displayed are percentages of CD19+ B cells (left panel: adult and right panel: neonates), and MZp/MZ B cells (middle panel: adult). (TIF) [file pone.0192230.s009.tif]
